# Intra‐arterial Delivery of Tislelizumab plus Transarterial Chemoembolization for Rectal Cancer: A Novel Regimen to Achieve Sphincter Preservation and Prevent Anastomotic Leakage

**DOI:** 10.1002/mco2.70456

**Published:** 2025-10-28

**Authors:** Wenjun Meng, Yueting Zhu, Jialing Wang, Jiadi Gan, Jiyan Liu, Dong Wang, Weimin Li, Chunxue Li

**Affiliations:** ^1^ Department of Pain Management West China Hospital Sichuan University Chengdu China; ^2^ Department of Biotherapy Cancer Center West China Hospital Sichuan University Chengdu China; ^3^ Department of Pulmonary and Critical Care Medicine West China Hospital Sichuan University Chengdu China; ^4^ Department of Oncology Chongqing University Qianjiang Hospital Qianjiang Central Hospital of Chongqing Chongqing China; ^5^ Department of General Surgery Daping Hospital Army Medical University Chongqing China

1

Dear Editor:

Rectal cancer (RC) is characterized by high morbidity and low quality of life (QoL), especially ultra‐low RC, which defines as the tumor located within 5 cm of the anal verge. Abdominoperineal resection (APR) is always regarded as the curative approach of ultra‐low RC. However, the biggest disadvantage of APR is the nonreversible pain that the permanent colostomy brings to the patient and the accompanying serious decline in QoL. To reach RC patients’ anus preservation as well as guarantee their mental health, multiple novel surgical approaches are proposed, such as low anterior resection (LAR), intersphincteric resection (ISR), coloanal anastomosis, and so on. However, the subsequent poor anal function after such conventional and advanced surgical methods seriously affects patients’ QoL. Studies have shown that ISR can lead to increased fecal incontinence and anal pain [1]. Additionally, conformal sphincter‐preservation operation is an extreme anus‐preserving operation for low RC, with a higher anal function and patient satisfaction compared with ISR [2]. However, due to the complex local tumor condition after neoadjuvant radiotherapy, surgery of sphincter preservation cannot be conducted in every patient with low RC. Nevertheless, no matter what surgical method is employed, anastomotic leakage (AL) cannot be completely avoided. This is because preoperative radiotherapy and surgery following the principle of total mesorectal excision can affect the blood supply and tissue healing ability of the anastomotic site, thereby increasing the incidence of AL. Apart from the injury of second operation due to AL, the occurrence of AL is also significantly associated with a greater risk of local recurrence and worse overall and cancer‐specific survival.

Another idea to avoid AL in RC patients is to furthest alleviate tumor burden by conducting efficient neoadjuvant therapy. In approximately 5% RC patients, whose DNA sequence belongs to mismatch repair deficiency or microsatellite instability high (MSI‐H), the use of immune checkpoint inhibitor (ICI) alone or in combination with chemoradiotherapy remains good results. However, the remain 90% RC patients are insensitive to ICIs, including antibodies of cytotoxic T lymphocyte‐associated protein 4, programmed cell death 1 (PD‐1), and programmed cell death ligand‐1 (PD‐L1), due to mismatch repair proficiency (pMMR) or microsatellite stable (MSS). RC patients with pMMR/MSS are marked by a reduced tumor mutational burden and scant T‐cell infiltration within the tumor microenvironment (TME). As a result, approaches that boost the tumor's ability to provoke an immune response, like chemoradiation, may hold promise for bettering the effectiveness of immunotherapy treatments targeting immune checkpoints for such patients [3].

To increase the efficacy of neoadjuvant ICI therapy in patients with RC, we proposed a phase 2 study (CIETAI study). In our study, preoperative loco‐infusion of tislelizumab and oxaliplatin to rectal tumor arteries followed by embolization with concurrent chemoradiotherapy serves as a novel neoadjuvant regimen to patients with locally advanced RC (LARC; T_3/4_N_any_M_0_ or T_1–4_N_+_M_0_). To the best of our knowledge, this study is the first to use ICI as local infusion drug in RC's neoadjuvant stage, and the treatment regimen and process are shown in Figure 1A. In this patient cohort, image‐guided chemo‐immuno‐embolization via rectal tumor arteries is conducted prior the standard neoadjuvant chemoradiotherapy. The detailed regimen of locoregional therapy is the infusion of oxaliplatin (130 mg/m^2^) and PD‐1 inhibitor tislelizumab (200 mg), followed by gelatin sponge particles (350–560 µm) embolization. To date, 27 patients have finished the preoperative treatment and following surgery, with a follow‐up time of at least ten months. The clinicopathological characteristics of the study patients are listed in Figure 1B. All of these LARC patients are of pMMR/MSS type. The tumor site from the anal verge ranged from 2.0 to 10.5 cm. Of these patients, four failed to undergo sphincter preservation surgery due to the high tumor grade after neoadjuvant therapy and poor local condition.

**FIGURE 1:**
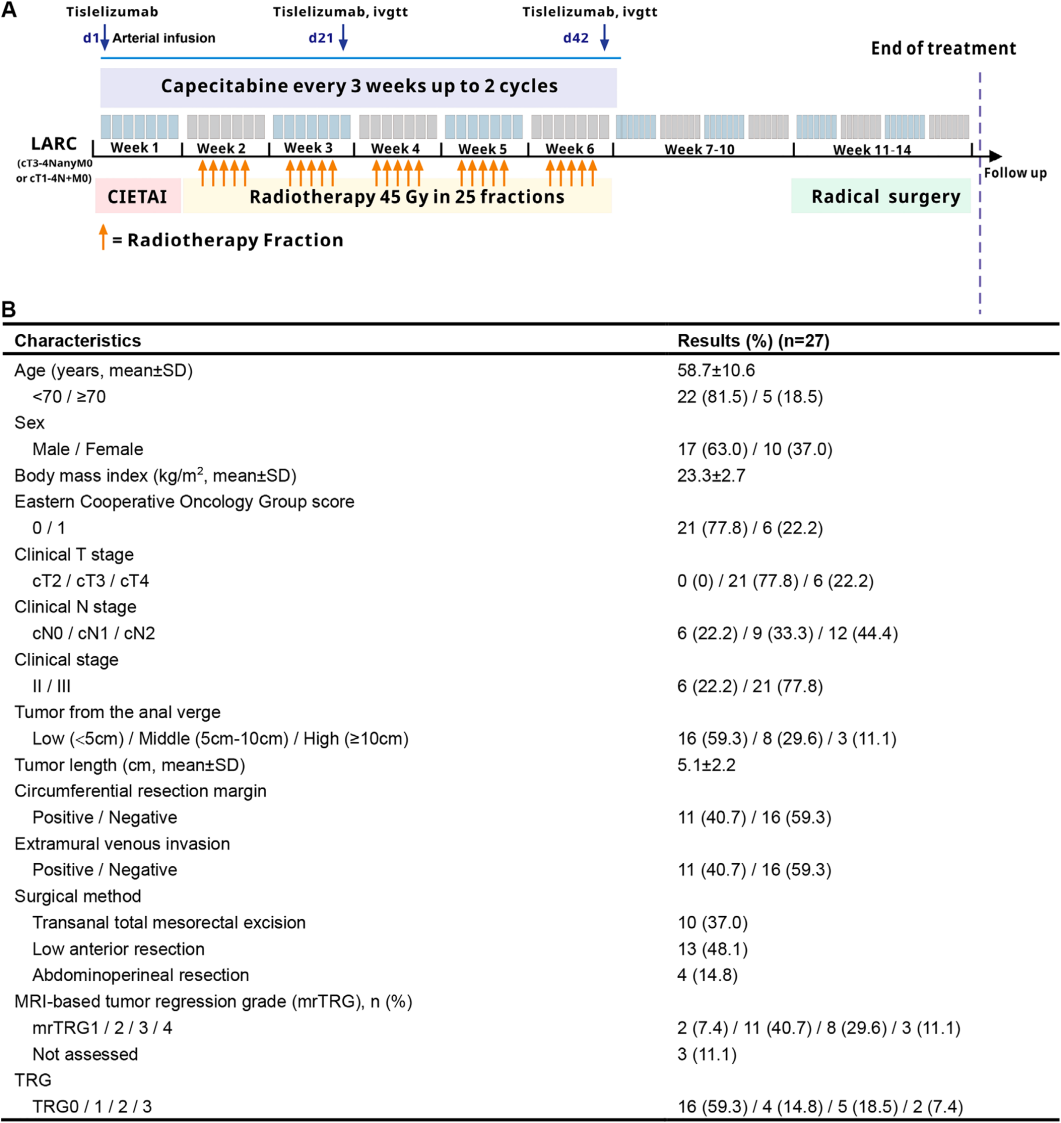
Overview of the study. (A) The treatment regimen and process. *Abbreviations*: CIETAI, chemo‐immuno‐embolization with transcatheter rectal arterial intervention; LARC, locally advanced rectal cancer. (B) Clinicopathological characteristics and therapeutic effects.

During the follow‐up, we mainly focus on the occurrence of AL as well as other postoperative complications. In these 27 patients, intestinal obstruction occurred in one patient, and fistula necrosis occurred in another patient undergone APR. No patients developed AL during at least 10 months’ follow‐up. The low AL probability indicated that the evaluation by multidisciplinary team was sufficient, and the interventional therapy had little impact on the local lesions. As a result, this novel treatment strategy reaches satisfactory results, especially in tumor relegation, sphincter preservation, as well as postoperative AL prevention.

As noted earlier, neoadjuvant radiotherapy increases the incidence of AL in RC patients, which is considered a double‐edged sword. Nevertheless, radiotherapy can enhance antitumor immune responses by promoting PD‐1/PD‐L1 expression in T‐cell of tumor tissues and the sensitivity to immunotherapy [3]. Furthermore, when combined with ICI, radiotherapy can modulate the TME and reduce its immunosuppressive effects [4]. By infusing PD‐1 inhibitor tislelizumab regionally in the tumor arteries, the above‐mentioned responses and sensitivity are further upgraded. In our study, by combining with intra‐arterial delivery, it may lead to better tumor shrinkage and improved tissue conditions, reducing tension on the anastomosis. Tislelizumab enhances immune activation, potentially promoting better healing and reduced local inflammation, which are crucial for anastomotic integrity. Furthermore, embolization selectively blocks larger tumor‐feeding arteries while preserving smaller ones, ensuring sufficient perfusion in surrounding tissues and preventing ischemia at the anastomotic site. Also, intra‐arterial drug delivery increases local concentration of chemotherapy while minimizing systemic side effects, which may contribute to better healing capacity of normal tissues and lower AL risk. As a result, it is being used to improve local drug concentration at the tumor site and enhance the immune response and synergize with chemoradiotherapy. Finally, the local tumor reached maximized reduction, offsetting the adverse effects of radiotherapy on operative region and anastomotic growth.

It is a great challenge to expand immunotherapy to pMMR/MSS subtypes in RC patients. For this subtype of patients, total neoadjuvant therapy (TNT) is offered in multiple guidelines as initial treatment especially in low RC patients. When clinical complete response (cCR) is reached after TNT, the watch‐and‐wait strategy can be considered. However, the small number of patients who eventually achieve cCR still leads to following surgery in most low RC patients. In TORCH study released recently, immunotherapy is added to TNT in pMMR/MSS LARC patients, showing a promising complete response rate with acceptable toxicity compared with traditional TNT [5]. TNT has its inevitable limitations, leading to its circumscribed range of applications. As a result, radical surgery is still the core method to achieve clinical cure. Immunotherapy has emerged as a promising strategy to enhance neoadjuvant therapy for LARC. Chemoradiotherapy promotes the release of tumor‐associated antigens, transforming immunologically noninflamed tumors into inflamed ones, thereby improving the response of MSS tumors to immunotherapy. In our study, tumor‐feeding artery embolization not only deprives tumor cells of essential nutrients but also prevents the systemic dispersion of anticancer agents, thereby increasing local drug concentration. This approach helps counteract the immune desertification commonly observed in rectal tumors and enhances their susceptibility to immunotherapy.

In conclusion, we first explored the feasibility of chemo‐immuno‐embolization with transcatheter arterial intervention in patients with RC. Our treatment strategy shows promising abilities to achieve sphincter preservation and prevent AL in patients with low LARC. Due to the small number in the current cohort, subsequent inclusion and follow‐up are needed to verify our protocol.

## Author Contributions

**Wenjun Meng**: conceptualization, data curation, and writing – original draft. **Yueting Zhu**: writing – original draft and funding. **Jialing Wang**: data curation and writing – review and editing. **Jiadi Gan**: writing – review and editing. **Jiyan Liu**: writing – review and editing and funding. **Dong Wang**: writing – review and editing and funding. **Weimin Li**: writing – review and editing and funding. **Chunxue Li**: conceptualization, writing – review and editing, supervision, and funding. All authors have read and approved the final manuscript.

## Conflicts of Interest

The authors declare no conflicts of interest.

## Ethics Statement

This study was approved by the Ethics Committee of Daping Hospital of Army Medical University (No. 2023–152), and registered on ClinicalTrials.gov (NCT05957016).

## Consent

Written informed consent was obtained from all participants.

## Supporting information




**Supporting File 1**: mco270456‐sup‐0001‐SuppMat.docx

## Data Availability

The data that supporting the study are available from the first author upon reasonable request.
