# Supplementary material for: Intra‐arterial Delivery of Tislelizumab plus Transarterial Chemoembolization for Rectal Cancer: A Novel Regimen to Achieve Sphincter Preservation and Prevent Anastomotic Leakage
Source: MedComm (2020). 2025 Oct 28;6(11):e70456. doi: 10.1002/mco2.70456 (PMC12559920; doi:10.1002/mco2.70456)
Supplement: Supplementary file 1 — Supporting File 1: mco270456‐sup‐0001‐SuppMat.docx [file MCO2-6-e70456-s001.docx]

**Intra-arterial delivery of Tislelizumab plus transarterial chemoembolization for rectal cancer: a novel regimen to achieve sphincter preservation and prevent** **anastomotic leakage**

**Running title:** Chemo-immuno-embolization for rectal cancer

Wenjun Meng^1#^, Yueting Zhu^1#^, Jialing Wang^1^, Jiadi Gan^1^, Jiyan Liu^1^, Dong Wang^2^, Weimin Li^1*^, Chunxue Li^2*^

**Affiliations:**

^1^ Department of Pain Management, West China Hospital, Sichuan University, Chengdu, China; Department of Biotherapy, Cancer Center, West China Hospital, Sichuan University, Chengdu, China; Department of Pulmonary and Critical Care Medicine, West China Hospital, Sichuan University, Chengdu, China

^2^ Department of General Surgery, Daping Hospital, Army Medical University, Chongqing, China; Cancer Center, Daping Hospital, Army Medical University, Chongqing, China

^#^These authors have contributed equally to this work

*** Corresponding author:**

Chunxue Li, Email: lichunxue_amu@163.com, Address: 10 Changjiang Zhi Road, Yuzhong District, Chongqing 400042, China

Weimin Li, Email: weimin003@163.com, Address: 37 Guoxue Lane, Wuhou District, Chengdu 610041, China

**Materials and Methods**

**Study design and participants**

This investigation is part of an ongoing, nonrandomized, single-arm phase II clinical trial conducted at Daping Hospital, Army Medical University. Eligible participants were individuals diagnosed with histologically confirmed, previously untreated rectal adenocarcinoma located within 15 cm from the anal verge, classified as clinical stage II or III (T_3-4_N_0_M_0_ or T_1-4_N_+_M_0_). Additional inclusion criteria required an Eastern Cooperative Oncology Group (ECOG) performance status of 0-1 and an age of 18 years or older. Patients were deemed ineligible if they met any of the following exclusion criteria: (a) presence of active autoimmune disorders or conditions necessitating immunosuppressive therapy; (b) contraindications to arterial puncture, immunotherapy, or chemoradiotherapy; (c) severe cardiopulmonary, renal, or hepatic dysfunction; (d) severe neurological or psychiatric disorders; or (e) pregnancy or lactation. Patient recruitment commenced in July 2023, and is scheduled for completion by June 30, 2026. Written informed consent was obtained from all participants, and the study protocol received approval from the Ethics Committee of Daping Hospital, Army Medical University (No. 2023-152). This trial is registered at ClinicalTrials.gov (NCT05957016) and adheres to the ethical standards outlined in the Declaration of Helsinki and Good Clinical Practice.

**Treatment procedures**

At baseline, participants underwent a comprehensive assessment, including full colonoscopy, pelvic magnetic resonance imaging (MRI), and computed tomography (CT) scans of the chest, abdomen, and pelvis to facilitate accurate staging. Baseline demographic and clinical data were also documented.

Following staging, eligible patients initiated CIETAI treatment. Procedural details were as follows: Initially, femoral artery puncture was performed to identify arterial supply to the rectal tumor. Subsequently, intra-arterial infusion of oxaliplatin (85 mg/m^2^) and PD-1 monoclonal antibody Tislelizumab (200 mg) was administered, targeting the tumor site. Embolization of the principal nutrient arteries was achieved using gelatin sponge particles and iodixanol, ensuring selective preservation of smaller tumor-feeding arteries to mitigate risks of tissue necrosis and perforation. Oxaliplatin dosing was calculated based on body surface area (130 mg/m^2^), with 100 mg diluted in 50 ml of 5% glucose solution infused via catheter, and the remaining dose administered intravenously. Concurrently, standard chemoradiotherapy (CRT) was implemented, comprising two 21-day cycles of capecitabine (1000 mg/m^2^, twice daily, orally, days 1-14) and radiotherapy delivering 45 Gy in 25 fractions. Additionally, tislelizumab (200 mg) was administered intravenously every three weeks for two additional cycles during CRT.

Following neoadjuvant therapy, patients underwent reassessment via colonoscopy, pelvic MRI, and CT imaging of the chest, abdomen, and pelvis to evaluate local and distant disease status. If metastatic lesions were suspected, 18F-fluorodeoxyglucose positron emission tomography (FDG-PET) was performed. Tumor regression was evaluated using the MRI-based tumor regression grading system (mrTRG) based on MERCURY trial criteria. The grading system categorized response as follows: mrTRG1 (complete regression, absence of tumor signal), mrTRG2 (good regression, predominance of fibrosis with no residual tumor signal), mrTRG3 (moderate regression, predominant fibrosis with some residual tumor signal), mrTRG4 (slight regression, minimal fibrosis with mostly tumor presence), and mrTRG5 (no regression, tumor signal identical to pre-treatment state). Current evidence suggests a strong correlation between mrTRG and patient prognosis in rectal cancer. Thus, mrTRG evaluation was mandated for all study participants.

Surgical resection was required and performed within 4 to 8 weeks following radiotherapy. Resected specimens were processed as formalin-fixed, paraffin-embedded (FFPE) tissue sections and assessed for pathological response per the American Joint Committee on Cancer (AJCC) 8th edition criteria. Tumor regression grade (TRG) was independently scored by two pathologists as follows: TRG0 (pathological complete response [pCR], no residual tumor), TRG1 (minimal residual cancer cells), TRG2 (residual cancer with evident regression), and TRG3 (extensive residual cancer with no significant regression). Major pathological response (MPR) was defined as the sum of TRG0 and TRG1, indicating a robust therapeutic response.

Adjuvant chemotherapy comprising mFOLFOX6 or CAPOX regimens was recommended for 4–6 months. Patients then entered a structured follow-up phase extending for three years, involving biannual assessments for the first two years and annual evaluations thereafter. Follow-up was conducted via telephone interviews and outpatient visits.

**Outcomes**

In this research letter, we mainly focus on the occurrence of anastomotic leakage (AL) as well as other postoperative complications during the follow-up. In our primary study protocol, the primary endpoint is pCR rate; the secondary endpoints include MPR rate, sphincter preservation rate, 3-year DFS, 3-year overall survival (OS), treatment-related adverse events (AEs) and surgical quality assessment.

**Statistical analysis**

Non-normally distributed data are represented as median with interquartile range, and normally distributed data were described as mean and standard deviation. Categorical data are presented as frequency and percentage. All statistical analyses were done using the SPSS software (SPSS Inc., Chicago, IL, version 26.0 for Windows).
